# Impact of *en bloc* extended R0 resections on oncological outcome of locally advanced adrenocortical carcinoma

**DOI:** 10.1007/s13304-025-02215-z

**Published:** 2025-05-03

**Authors:** Priscilla Francesca Procopio, Francesco Pennestrì, Antonio Laurino, Esther Diana Rossi, Giovanni Schinzari, Alfredo Pontecorvi, Carmela De Crea, Marco Raffaelli

**Affiliations:** 1 U.O.C. Chirurgia Endocrina E Metabolica, Fondazione Policlinico Universitario Agostino Gemelli IRCCS, Rome, Italy; 2 Centro Di Ricerca in Chirurgia Delle Ghiandole Endocrine E Obesità, Università Cattolica del Sacro Cuore, Rome, Italy; 3 U.O.C. Anatomia Patologica Della Testa E Collo, Fondazione Policlinico Universitario Agostino Gemelli IRCCS, del Polmone E Dell’Apparato Endocrino, Rome, Italy; 4 Dipartimento Di Scienza Della Vita E Sanità Pubblica, Università Cattolica del Sacro Cuore, Rome, Italy; 5 Comprehensive Cancer Center, U.O.C. Oncologia Medica, Fondazione Policlinico Universitario Agostino Gemelli IRCCS, Rome, Italy; 6 Dipartimento Di Medicina E Chirurgia Traslazionale, Università Cattolica del Sacro Cuore, Rome, Italy; 7 Endocrinologia E Diabetologia, U.O.C. Medicina Interna, Fondazione Policlinico Universitario Agostino Gemelli IRCCS, Rome, Italy

**Keywords:** Adrenocortical carcinoma (ACC), ENSAT Stage III, Extended multiorgan resection, Locoregional recurrence, Oncological outcome, R0 resection

## Abstract

In locally advanced adrenocortical carcinoma (ACC) (ENSAT stage III – S-III) R0 surgery, involving *en bloc* extended resections, is the only potential curative treatment. We evaluated oncological outcomes and complications rate in S-III patients who underwent extended resection in comparison with stage I/II (S-I/II). Among 1098 adrenalectomies over 27 years (1997 –2024) in a tertiary referral center, medical records of ACC patients were reviewed, excluding stage IV and not-multivisceral resections in S-III patients. Forty-eight patients met the inclusion criteria: 6 S-I (12.5%), 36 S-II (75%) and 6 S-III (12.5%) patients. The latter patients’ cohort underwent multivisceral *en bloc* resections (3 total nephrectomies, one renal vein thrombectomy, one splenopancreasectomy associated with total nephrectomy, left hemicolectomy and omentectomy, one liver S6-S7-S8 resection). Open adrenalectomy was scheduled in all S-III patients. Minimally-invasive approach was scheduled in 21 (50%) S-I/II patients. Conversion to open adrenalectomy was registered in 5 out these 21 patients. Locoregional and distant disease recurrences were registered in 19% of S-I/II vs 33.3% of S-III patients and 28.6% of S-I/II vs 66.7% of S-III patients, respectively (*p* = 0.420, *p* = 0.064). Postoperative complications were observed in 21.4% of S-I/II patients and 16.7% of S-III patients (*p* = 0.788). Kaplan–Meier DFS and OS curves were comparable among the two groups (*p* = 0.255, *p* = 0.459, respectively). After univariable analysis, hyperfunction and chemotherapy were significantly associated with locoregional disease recurrence (*p* = 0.02, *p* = 0.04, respectively). OS and DFS of S-III ACC patients undergoing extended *en bloc* R0 resections were comparable to those of S-I/II patients, without increased postoperative morbidity.

## Introduction

Adrenocortical carcinoma (ACC) is a rare neoplasm, affecting 0.7 to 2 patients per million per year, with a dismal prognosis, being the second most aggressive endocrine malignancy behind undifferentiated thyroid cancer [1, 2].

Prognosis in ACC is mainly based on tumor stage (S), and the outcomes may be widely heterogeneous [3, 4]. In this view, the 5-years overall survival (OS) has been strictly related to ENSAT staging system, dropping from 82% (69%−99%) in S-I (≤ 5 cm) tumors to 61% (51%−69%) in S-II (> 5 cm) tumors and 50% (39%−61%) in S-III tumors (with positive lymph nodes and/or extra-adrenal tissue infiltration and/or venous thrombus in renal vein/inferior vena cava-IVC) [5–8].

Despite standardization of adjuvant treatment, prognosis did not significantly change in the last decades [8]. This evidence may be partially explained by ACC high propensity to relapse [5]. Both locoregional and distant disease recurrence is associated to a more aggressive biologic behavior, thus more difficult to control [5]. As a consequence, ACC recurrence deeply impacts on OS as well as on patients’ quality of life [5].

To date, therapeutic options for ACC are still limited [5]. The lack of significant improvements in medical treatment comparing to the majority of solid tumors is not surprising, given the rarity of the disease and the paucity of the available resources to improve therapeutic strategies [5].

The only chance for cure in patients without metastatic disease is complete primary tumor resection, avoiding effraction of the tumor capsule or spillage of neoplastic cells and achieving microscopically margin-free resection (R0) [9, 10]. Indeed, an incomplete surgical treatment in terms of resection status impairs the definitive survival, as R1 and R2 (defined as microscopic and macroscopic residual disease on margins, respectively) are associated with an increased risk of death compared to R0 margins [5].

Oncological radical resection of adrenal tumor must include *en bloc* surgical removal of periadrenal fat or even further organs or anatomic structures in case of evidence of infiltration [11]. Lymph node dissection (LND) is suggested by the ESES/ENSAT recommendations with both therapeutic and better staging aim [9, 12]. Such recommendation relies on the evidence that regional lymph node involvement in ACC has a negative impact on OS and is frequently the cause of locoregional recurrence [9, 13–15]. Indeed, in absence of an accurate LND, nodal status cannot be appropriately evaluated and S-III ACCs could potentially be undertreated [12]. At the same time, metastatic lymph node involvement is associated with incomplete resection status due to the missed dissection of the tumor lymphatic drainage, leading to inferior outcomes [12].

The present study aims to evaluate the oncological benefit of R0 resections for locally advanced disease by comparing the clinical outcomes of S-I and S-II patients, who underwent adrenalectomy, with S-III patients, who underwent extended *en bloc* surgical resections.

## Material and methods

### Study design

This is a retrospective cohort study considering patients who underwent adrenalectomy and received pathologic diagnosis of ACC between January 1997 and October 2024 in our national referral center for endocrine surgery.

Data have been prospectively collected in a specifically designed de-identified electronic database. Patients’ follow-up was conducted until 30^th^ November 2024.

All surgical operations of suspicious adrenal lesions and ACC recurrences have been performed by an experienced endocrine surgeon [16, 17].

Inclusion criteria consisted in adult (≥ 18 years-old) patients who underwent index R0 operation in our center for adrenal lesions with diagnosis of ACC after pathologic examination.

Exclusion criteria were: S-IV disease, operations for disease recurrence in patients who underwent index surgery in other centers, S-III patients who did not undergo multivisceral *en bloc* surgical resections in association to adrenalectomy plus LND and postoperative follow-up < 6 months.

Preoperative data included demographic characteristics (age, BMI, sex as assigned at birth), hormonal hyperfunction, lesion side and size. Operative parameters included *intention to treatment* surgical technique (open and minimally-invasive surgery), *intention to treatment* surgical approach to and *per protocol* adrenalectomy, including open adrenalectomy, converted adrenalectomy, laparoscopic lateral transperitoneal adrenalectomy (LTA), posterior retroperitoneoscopic adrenalectomy (PRA) and robot-assisted adrenalectomy (RAA) by lateral transperitoneal approach. Conversion rate, LND, extended *en bloc* surgical resections associated to adrenalectomy and operative time were also considered. Postoperative variables included postoperative intensive unit care stay, early (within 30 days) and late postoperative complications, lesion’s pathologic features (size, histotype, resection status and Ki-67 index—when available), adjuvant therapy (including mitotane, chemotherapy and radiotherapy), locoregional and distant recurrence of the disease, date of the last follow-up or death.

Adjuvant therapy, including mitotane and/or cytotoxic agents (EDP scheme—etoposide, doxorubicin and cisplatin), was administrated in selected cases after accurate multidisciplinary evaluation of lesions and patients features. Patients were addressed to adjuvant radiotherapy in case of risk factors for recurrence, such as high lesion’s dimension, grading (Ki-67 index) and pT4 disease.

Patients were divided into two groups: S-I/II patients and S-III patients.

The primary outcome consisted in the comparison of oncologic outcomes in terms of locoregional recurrence of the disease between S-I/II patients, who underwent adrenalectomy, with S-III patients, who underwent extended *en bloc* surgical resections.

The secondary outcomes of the study were the evaluation of DFS, OS and early postoperative complications rate. A subgroup analysis was also performed to evaluate locoregional recurrence-related factors.

The study was approved by the Ethical Committee of our Center (ID 5171).

### Definitions and surgical planning

Assessment of preoperative work-up was based on guidelines provided by the National Institutes of Health and recommendations from the ENSAT/ESES societies concerning the care of ACC patients [18–20].

The aim of the surgical treatment was achieving R0 resection status at pathologic examination.

Surgical plan was designed as adrenalectomy alone vs multiorgan resection (adrenal plus at least one or more adjacent organs and/or venous thrombectomy) basing on pre- and/or intraoperative evidence or suspect of extra-adrenal disease extension. Resection status was defined as R0, R1 or R2 after pathologic examination [18]. LND was performed in both groups basing on pre- and intraoperative suspect of lymph node involvement and/or according to different suggestions through the years [9]. LND included as a minimum the periadrenal and renal hilum nodes [9]. In selected cases further nodal dissection included celiac trunk, para-aortic, interaortocaval, retropancreatic, retrocaval, diaphragmatic, right and common hepatic and hepatic hilum nodes.

Surgical procedures included open adrenalectomy, LTA, PRA and RAA, as previously described in details [21].

Operative time is defined as the interval from incision to wound closure (skin to skin). In case of robotic surgeries, operative time also included docking time.

The severity of postoperative complications was graded according to the Clavien-Dindo classification [22].

Disease recurrence was diagnosed based on clinical, laboratory results and/or radiological evaluation. Locoregional recurrence has been defined as recurrence of the disease at the surgical site (adrenal lodge or locoregional lymph nodes) [9, 23], while recurrence in other anatomical regions has been defined as progression of systemic disease.

The period of DFS was calculated from the date of the surgical procedure to the last follow-up evaluation of patients without recurrence. The period of OS for the study was calculated from the date of the surgical procedure to the date of death or of the last follow-up for surviving patients.

### Statistical analysis

Baseline characteristics and perioperative variables were compared using a bivariable analysis. Normal distribution was assessed using the Shapiro-Wilks test. Chi-square test was used to compare categorical variables. Continuous variables were expressed as median (interquartile range, IQR). We used paired sample t test or Wilcoxon test to compare continuous variables, depending on data distribution of the analyzed population. Backward stepwise logistic regression (multivariable analysis) was performed to evaluate the potential risk factors for locoregional recurrence. At each step, the variable that had the lowest correlation with the outcome was removed with an elimination criterion set at *p* > 0.100 and a threshold of *p* = 0.1 to set a limit on the total number of variables included in the final model. Only variables with a p < 0.2 on univariable analysis were entered in the model.

DFS and OS curves were calculated according to the Kaplan–Meier method and were compared by means of the log-rank test.

Statistical analysis was conducted with SPSS 22.0 software for Windows (SPSS Inc, Chicago, III). MedCalc 18.2.1 (MedCalc Software Ltd, Ostend, BE) was used for survival analysis. All analyses were two tailed, and the threshold for statistical significance was set at p < 0.05.

## Results

Among 1098 adrenalectomies performed between January 1997 and October 2024, we selected 65 patients with pathologic diagnosis of ACC.

Three patients were excluded because they only underwent surgical resection of disease recurrence in our center, while index operation of ACC was performed in other hospitals. Eleven patients were excluded due to ENSAT S-IV (oligometastatic disease) at time of diagnosis. In these patients, surgical resection of ACC was scheduled to improve clinical management of hypercortisolism-related symptoms. Three further patients were excluded as adrenalectomy was not associated to extended multivisceral *en bloc* surgical resections, with definitive pathology reporting incidental diagnosis of S-III disease. Overall, 48 patients met the inclusion criteria: 42 S-I/II patients (including 6 S-I patients and 36 S-II patients) and 6 S-III patients.

In Table 1, details concerning disease staging of S-III patients are provided *in extenso*.

**Table 1:** *En bloc* extended surgical resections for S-III patients

TNM	Lymph node dissection	Surgical resections associated to adrenalectomy
pT3 N0	Renal hilum	Total nephrectomy
pT3 N0	Renal hilum	Total nephrectomy
pT3 N0	Renal hilum	Total nephrectomy
pT3 N1 (venous thrombus)	Renal hilum Celiac Trunk	Renal vein thrombectomy
pT2 N1	Renal hilum Celiac trunk Para-aortic Retropancreatic Aorto-caval	Total nephrectomy Splenopancreasectomy Left hemicolectomy Omentectomy
pT4 N0	Renal hilum Aorto-caval Common hepatic artery Right hepatic artery Diaphragmatic	Liver S6-S7-S8 resection

In Table 2, we compared the main perioperative variables of the included patients.

**Table 2:** Comparison between S-I/II and S-III patients – main preoperative, operative and postoperative variables

	Stage	Stage	*p*-value
	I/II	III	
Patients	42	6	–
Age (years)	50.5 (39—65)	48.5 (45.7—64.2)	0.869
BMI (Kg/m^2^)	24.1 (22.4—27.8)	24.4 (20.5—24.4)	0.91
Sex			
Male	17 (40.5%)	1 (16.7%)	0.26
Female	25 (59.5%)	5 (83.3%)	
Hyperfunction			
No	29 (69.0%)	3 (50.0%)	0.355
Yes	13 (31.0%)	3 (50.0%)	
Side			
Right	19 (45.2%)	2 (33.3%)	0.582
Left	23 (54.8%)	4 (66.7%)	
Technique			
Open	21 (50.0%)	6 (100%)	0.021
Minimally-invasive	21 (50.0%)	0 (%)	
Approach			
Open	21 (50.0%)	6 (100%)	0.149
TLA	13 (31.0%)	0 (0%)	
PRA	1 (2.4%)	0 (0%)	
RA	7 (16.6%)	0 (0%)	
Adrenalectomy			
Open	21 (50.0%)	6 (100.0%)	0.255
Conversion	5 (12.0%)	0 (0%)	
TLA	10 (23.9%)	0 (0%)	
PRA	1 (2.3%)	0 (0%)	
RAA	5 (11.9%)	0 (0%)	
Lymph node dissection			
No	30 (71.4%)	0 (0%)	0.001
Yes	12 (28.6%)	6 (100%)	
Extended resection			
No	37 (88.1%)	0 (0%)	< 0.001
Yes	5 (11.9%)	6 (100%)	
Operative time (minutes)	105 (84.2—154.7)	236 (135.5—336.5)	0.008
Postoperative Intensive Care			
No	41 (97.6%)	5 (83.3%)	0.101
Yes	1 (2.4%)	1 (16.7%)	
Postoperative complications			
No	33 (78.6%)	5 (83.3%)	0.788
Yes	9 (21.4%)	1 (16.7%)	
Tumor size (mm)	92 (60 −128.5)	110 (71.2—185)	0.34
Postoperative hospital stay (days)	5 (3.5—8)	9 (4–10.5)	0.449
Histotype			
Classic variant	29 (69.0%)	4 (66.7%)	0.096
Oncocytic variant	13 (31.0%)	2 (33.3%)	
Adjuvant therapy			
No	12 (28.6%)	0 (0%)	0.131
Yes	30 (71.4%)	6 (100%)	
Mitotane			
No	13 (31.0%)	1 (16.7%)	0.471
Yes	29 (69.0%)	5 (83.3%)	
Chemotherapy			
No	34 (81.0%)	4 (66.7%)	0.42
Yes	8 (19.0%)	2 (33.3%)	
Radiotherapy			
No	39 (92.9%)	5 (83.3%)	0.43
Yes	3 (7.1%)	1 (16.7%)	
Locoregional recurrence			
No	34 (81%)	4 (66.7%)	0.42
Yes	8 (19%)	2 (33.3%)	
Distant recurrence			
No	30 (71.4%)	2 (33.3%)	0.064
Yes	12 (28.6%)	4 (66.7%)	
Follow-up time (months)	44 (19.2—96)	19 (13.2–137.5)	0.691

Continuous variables are expressed as median (IQR)

Preoperative features, including demographic characteristics, hyperfunction and lesion side were comparable among the two groups.

Open adrenalectomy was scheduled in all S-III patients. Minimally-invasive approach was scheduled in 21 (50%) S-I/II patients. Conversion to open adrenalectomy was registered in 5 out these 21 patients, justified by tight adhesion due to previous abdominal surgeries (one case) and intraoperative suspect of extra-adrenal extension of the disease (four cases). A detailed description of surgical extent of S-III patients is also reported in Table 1. Multivisceral resections were also performed in 5 S-II patients, consisting in three partial nephrectomies, one total nephrectomy and one splenectomy, due to the intraoperative suspect of focal extension of the disease, with the aim of guarantying R0 resection margins.

Overall, a significant higher rate of S-III patients underwent multivisceral *en bloc* resections comparing to S-I/II patients: 100% vs 11.9%, respectively (*p* < 0.001).

LND was also performed significantly more often in S-III group (100% S-III patients vs 28.6% S-II patients, *p* = 0.001).

Median operative time was significantly shorter in S-I/II group (105 vs 236 min, *p* = 0.008).

The two groups were comparable in terms of postoperative intensive care stay, hospital stay and postoperative complications. In details, postoperative complications were registered in 10 out of 48 patients of the series: 9 (21.4%) S-I/II patients vs 1 (16.7%) S-III patients (*p* = 0.788). Minor complications (≤ II grade according to Clavien–Dindo classification [22]) occurred in both groups of patients, consisting in pneumonia (3 S-I/II patients and one S-III patient), wound infection (2 S-I/II patients), urinary infection (2 S-I/II patients) and intra-abdominal collection (1 S-I/II patient). Only one S-I/II patient experienced a major complication for intra-abdominal bleeding, which required reoperation (Clavien–Dindo grade IV[22]).

All surgical resections were confirmed R0 after definitive pathologic evaluation.

Median lesion size was higher in S-III group, though not statistically so (110 vs 92 mm, *p* = 0.340). Histotype was also evaluated for both groups, with no significative differences in terms of classical and oncocyte variants. Delving deeper, classical histotype was found in 69% of S-I/II patients and in 66.7% of S-III patients, while oncocyte variant in 13% of S-I/II patients and in 33.3% of S-III patients (*p* = 0.096).

No significant differences were found between the two groups in terms of adjuvant treatments (including mitotane, chemotherapy and radiotherapy).

Comparable locoregional recurrences were reported in the two groups: 8 out of 42 SI-II patients (19%) vs 2 out of 6 S-III patients (33.3%) (*p* = 0.420). A slight difference in terms of distant recurrences was registered, though not statistically significant: 12 out of 42 SI-II patients (28.6%) vs 4 out of 6 S-III patients (66.7%) (*p* = 0.064).

Moreover, 2 patients in each group presented both locoregional and distant recurrence of the disease.

Despite no statistically difference was recognized, we registered median shorter follow-up for S-III patients: 19 (13.2–137.5) months in S-III group vs 44 (19.2–96) months in S-I/II group (*p* = 0.691).

**Fig. 1:**
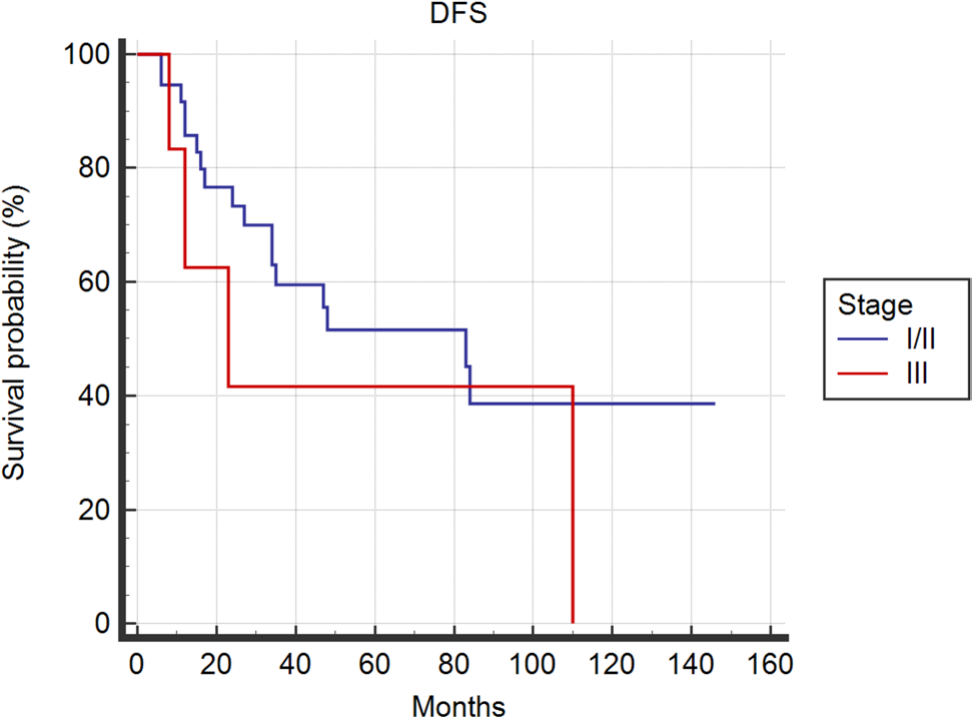
Kaplan––Meier Curves for Disease Free Survival of Stage I/II and Stage III ACC patients

**Fig. 2:**
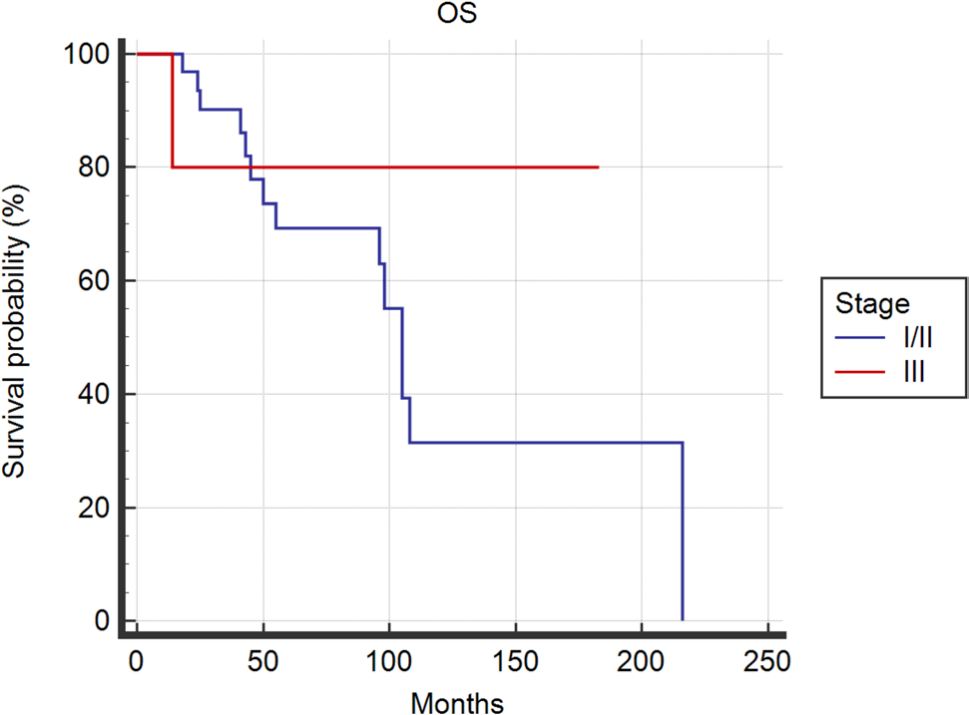
Kaplan––Meier Curves for Overall Survival of Stage I/II and Stage III ACC patients

Kaplan––Meier DFS and OS curves were similar among the two groups by means of log-rank test (*p* = 0.255, *p* = 0.459, respectively) (Fig. 1, Fig. 2). Delving deeper, 5-years-DFS and 5-years-OS were 49.6% vs 47.9% and 69.2% vs 69.6% in S-I/II group and S-III group, respectively.

After univariable analysis, hyperfunctioning lesions and adjuvant administration of cytotoxic treatments were significantly associated with locoregional recurrence of the disease (*p* = 0.02, *p* = 0.04, respectively) (Table 3).

**Table 3:** Univariable analysis for locoregional recurrence of disease

	Locoregional recurrence	Locoregional recurrence	*p*-value
	NO	YES	
	*n* = 38	*n* = 10	
Stage			
I/II	34 (89.5%)	8 (80.0%)	0.788
III	4 (10.5%)	2 (20.0%)	
Age (years)	51 (39–64.2)	58.5 (41.2–78.7)	1
Sex			
Male	16 (42.1%)	2 (20.0%)	0.199
Female	22 (57.9%)	8 (80.0%)	
BMI (Kg/m^2^)	25.3 (22.3–29.3)	23.6 (21.5–24.3)	0.315
Hyperfunction			
No	29 (76.3%)	3 (30.0%)	0.006
Yes	9 (23.7%)	7 (70.0%)	
Side			
Right	16 (42.1%)	6 (60.0%)	0.654
Left	22 (57.9%)	4 (40.0%)	
Approach			
Open	22 (57.9%)	5 (50.0%)	0.537
Conversion	3 (7.9%)	2 (20.0%)	
Minimally-invasive	13 (34.2%)	3 (30.0%)	
Lymph node dissection			
No	23 (60.5%)	7 (70.0%)	0.582
Yes	15 (39.5%)	3 (30.0%)	
Operative time (minutes)	117.5 (84.2–145)	179.5 (78.7–252.5)	0.505
Postoperative complications			
No	31 (81.6%)	7 (70.0%)	0.422
Yes	7 (18.4%)	3 (30.0%)	
Tumor size (mm)	92 (47.2–130)	123.5 (90–159.2)	0.673
Histotype			
Classic variant	25 (65.8%)	8 (80.0%)	0.388
Oncocytic variant	13 (34.2%)	2 (20.0%)	
Mitotane			
No	10 (26.3%)	4 (40.0%)	0.397
Yes	28 (73.7%)	6 (60.0%)	
Chemotherapy			
No	33 (86.8%)	5 (50.0%)	0.011
Yes	5 (13.2%)	5 (50.0%)	
Radiotherapy			
No	34 (89.5%)	10 (100.0%)	0.284
Yes	4 (10.5%)	0 (0.0%)	

Continuous variables are expressed as median (IQR)

No independent risk factors for locoregional recurrence of the disease were identified after multivariable analysis.

## Discussion

This study supports the surgical oncological effectiveness of extended resections of locally advanced ACCs, reporting similar results in terms of locoregional recurrence compared to patients with disease confined to the adrenal gland.

ACC has to be tackled with a limited arsenal of therapeutic options of which surgery remains the first choice [5]. *En bloc* complete resection of ACC with the peritumoral/periadrenal fat in absence of capsular effraction is considered the most important component of treatment with curative intent, as is the only chance for long-term cure [2, 5, 9]. Indeed, incomplete surgical radicality relates to increased risk of cancer-related death [5].

Application of neoadjuvant treatment to locally advanced ACC to achieve downsizing/downstaging to potentially allow R0 resection is still not standardized [5, 24, 25]. Similarly, efficacy of chemotherapy in the adjuvant setting is still debated, mainly due to ACC poor response to cytotoxic treatment [26, 27]. To date, ESES/ENSAT 2018 recommendations [9] did not reach consensus on this concern, though panelists suggest that they may be considered in selected patients with very high risk for recurrence. To date, mitotane still remains the only medication specifically approved for ACC. Adjuvant early administration of mitotane has been related to improved DFS and OS in advanced disease [3, 11] and it is currently mainly proposed basing on risk factors for disease recurrence, such as Ki-67 ≥ 10%, Rx/R1 resection status and high stage (S-III/IV), balancing them with toxic side effects [4, 11]. Clinical trials such as FIRM-ACT [27] highlighted the efficacy of combination of EDP with mitotane, with a high rate of objective tumor response and a significant increased progression-free survival when compared to combination of streptozocin plus mitotane. However, this trial patients’ cohort only included those ones with diagnosis of ACC not amenable to radical surgical resection [27]. Moreover, ACC has been historically considered a radioresistant disease. Retrospective studies on adjuvant use of radiotherapy in ACC have shown no beneficial effects [26, 28].

As a consequence of the aforementioned considerations, management of locally advanced ACC patients is known to be challenging. Indeed, literature reports suggest that R+ surgery and ENSAT S-III disease represent risk factors for disease recurrence compared to oncological radicality of surgical resection and early ACC stage [5]. Obviously, S-III patients undergoing less than extended surgical resections theoretically present a higher risk of R+ surgery and/or infiltrated margins at pathologic examination.

With regard to the primary endpoint of our study, our results show comparable locoregional recurrence rates between the two groups, with 8 S-I/II and 2 S-III patients developing locoregional disease recurrence (p = 0.420).

However, even though R0 resection has been widely defined as the only effective therapeutic strategy [5, 29], literature data on recurrence rate in locally advanced disease are still lacking. A recent ESES panel [29] expressed against nephrectomy in association to adrenalectomy to achieve oncological favorable results, in absence of pre- and intraoperative evidence of extra-adrenal extension of the disease. On the other hand, they reported encouraging outcomes after extended resections due to ACC infiltration of IVC, although such surgical strategies should be demanded in high-expertise hands and in referral centers with multidisciplinary management of the disease [30].

In this context, in our experience, achieving R0 resection margins and avoiding capsular effraction were deemed to be essential to considerably affect the natural history of the disease. Indeed, intraoperative suspect of extra-adrenal extension guided surgical planning even in case of lack of preoperative evidence of infiltration, as definitive pathologic examination represents the only means to confirm disease stage.

Moreover, to date, there is no clear consensus about the extension of LND in ACC [31]. As a consequence, as long as there is no surgical standard, its exact diagnostic value as well as the oncological effect cannot be still clearly assessed [31]. Discrepant reports regarding lymph node involvement ranging from 4 to 73% [2, 7, 32] suggest that formal regional lymphadenectomy is neither properly performed by surgeons nor accurately assessed or reported by pathologists [9]. Furthermore, retrospective data suggest that regional lymph node involvement in ACC negatively impact on OS and is frequently the cause of locoregional recurrence [6, 12, 14]. Concordantly with such literature evidence, we reported a slightly higher rate of locoregional recurrence in patients who did not undergo LND in association to the index operation, though not statistically significative (as reported in Table 3).

The lack of standardization of lymphadenectomy in ACC may also explain the difference of OS and DFS between S-II and S-III patients, as it can be related to understaging thus to undertreatment of S-III patients in adjuvant setting [12].

Nonetheless, our results concerning 5-years-OS and DFS are similar with literature reports [33, 34], being even superior compared to data reported from other authors’ experience [35], thus underlining R0 resection as the critical prognostic factor.

It may be argued that OS and DSF in S-I/II patients could have been affected by the high rate of minimally-invasive surgeries. However, we reported an equal distribution of locoregional recurrences between open (five cases), converted (two cases) and minimally invasive (three cases) adrenalectomies. Thus, surgical approach did not represent a risk factor for disease locoregional recurrence.

Indeed, although, in the beginning of laparoscopic application to ACC surgery, minimally invasive approach was associated with a higher frequency of R+ surgery and intra-abdominal recurrences, more recently several meta-analyses did not report impaired oncological outcomes after endoscopic treatment [5, 31, 36–38]. Hence, the choice of the best surgical approach for ACC or suspected malignant adrenal mass should be tailored on preoperative lesion’s features and evidence of local invasion to achieve a complete resection of the tumoral mass *en bloc* with the periadrenal fat to avoid tumor or capsular rupture or spillage [2, 5, 9]. Indeed, even if guidelines from two European societies [9, 39] suggest that potentially malignant adrenal tumors < 6 cm without invasion of adjacent organs may be eligible to minimally invasive adrenalectomy, several authors stated that endoscopic approach may not be excluded even in case of preoperative suspect of S-III disease, when surgery is performed in experienced hands and in high-volume centers [40, 41]. Interestingly, more recently Olivero et al. [40] reported clinical outcomes of patients with ACC with venous thrombus extension treated with both open and minimally invasive approaches, without significative differences among the two groups.

Moreover, the most recent ESES expert opinion [9] described robotic platforms as potential surgical alternative to other minimally invasive approaches even for the treatment > 6 cm lesions, due to their superiority in terms of three-dimensional vision, dexterity and stability, thus minimizing the risk of capsular rupture or R+ margins [39, 41], suggesting that RAA may be the preferred technique if a minimally invasive procedure is pursued [41].

Postoperative complications represent one of the main concerns related to extended surgical resections. Our analysis shows comparable results between the two groups (21.4% of S-I/II patients vs 16.7% of S-III patients, p = 0.788), in line with literature data resulting from meta-analysis, ranging from median values of 19.4% to 21.4% [38].

Lastly, we made the effort to try to identify potential risk factors for disease locoregional recurrence, though with the limitation of the relatively small sample size dimension. After univariable analysis, hyperfunctioning lesions were related to locoregional recurrence (p = 0.006; Odds Ratio 11.7 – Interval of Confidence 95%: 2–66.4), in line with literature reports on their association with increased biological aggressiveness [4]. Administration of adjuvant cytotoxic treatments also significantly related to disease locoregional recurrence (*p* = 0.011; Odds Ratio 8.5 – Interval of Confidence 95%: 1.7–42.8). Such evidence is not surprising, though apparently paradoxical, as it should be explained by the correct identification of preoperative suspicious lesion’s features of those patients who could have benefit adjuvant treatment the most, despite its aforementioned limited efficacy. In this view, adjuvant therapy should be considered as a predictor factor rather than a risk factor for locoregional disease recurrence. These considerations may also explain the higher rate of distant disease recurrence in S-III patients’ group, as a results of increased biological aggressiveness and unresponsiveness to systemic treatments.

The main limitations of the study are due its monocentric and retrospective nature over a long period involving a relative limited number of patients. However, this aspect should be correlated to the rarity of the disease. On the other hand, the strength of our results relies on the high-volume experience of our referral center, with a case volume of 90 adrenalectomies per year, and the multidisciplinary management of the disease. The role of the high volume of the center has been underlined by different societies consensus and recommendations [9, 16, 19, 29]. In this context, more than ten years ago, we reported one of the preliminary experiences in literature concerning the correlation between center’s volume and oncological outcomes [18]. These results have also been reported in larger retrospective series from MD Anderson Cancer Centre and in a Dutch series, with longer OS for S-I/III patients [31, 32, 42, 43].

Moreover, as far as we know, our reported experience represents the first study which compares oncological outcomes of S-III patients, treated with extended surgical resections, with S-I/II patients.

In conclusion, despite the limitations of the lacking effectiveness of systemic treatment in the adjuvant setting, R0 surgical resection seems to relate to improved oncological outcomes of locally advanced ACC.

However, further studies with larger sample size are still necessary to draw definitive conclusions.

## Data Availability

The raw data supporing the conclusion of this article will be made available by the corresponding author, without undue reservation.
